# Improved access to the innovative anticancer therapies in resource-limited countries: call for global action

**DOI:** 10.1097/JS9.0000000000001413

**Published:** 2024-03-27

**Authors:** Yosra Vaez-Gharamaleki, Mohammad-Salar Hosseini

**Affiliations:** a Hematology and Oncology Research Center, Tabriz University of Medical Sciences; b Student Research Committee, Tabriz University of Medical Sciences; c Research Center for Evidence-Based Medicine, Iranian EBM Center: A JBI Center of Excellence, Tabriz University of Medical Sciences; d Research Center for Integrative Medicine in Aging, Aging Research Institute, Tabriz University of Medical Sciences; e Clinical Research Development Unit of Tabriz Valiasr Hospital, Tabriz University of Medical Sciences, Tabriz, Iran

*Dear Editor,*

Cancer remains a global health challenge, with an overwhelming increase in the incidence and prevalence in recent decades, pushing health systems and scientists to propose more effective strategies for cancer management^1^. Innovative anticancer therapies, such as novel immunotherapies, protein kinase inhibitors, monoclonal antibodies, and antimetabolic drugs – either as mono- or combination therapy – have shown promising advancements in cancer treatment, exhibiting solid potential in enhancing therapeutic efficacy and improving patient outcomes^2^. Recent evidence unravels ‘dramatic disparities’ in treatment outcomes among people with cancer globally, part of which is undeniably due to disparities in access to efficient treatments^3,4^. MohanaSundaram *et al*.^5^ have recently addressed critical issues with medication provision in low- and middle-income countries. We aim to delve deeper into the disparities affecting the health outcomes of patients in low- and middle-income countries, either by lack of specialties, failure of interprofessional collaboration, substandard medications, or, most importantly, lack of access to effective treatments^6^.

The significance of promoting access to innovative anticancer therapies and medications is particularly pronounced in resource-limited countries, where life-limiting diseases, such as cancer, pose a double burden on the healthcare systems^7^. These nations often face barriers to the adoption of recently developed treatment strategies due to economic constraints, limited healthcare infrastructure, and disparities in research and development investments. Addressing this issue is essential, given the potential of innovative anticancer drugs to revolutionize treatment outcomes and enhance the patients’ quality of care and survival rates^8^.

There are several aspects to this issue, most important of them being:

1. Health disparities: Resource-limited and developing countries often face disparities in healthcare provision, exacerbating the challenges associated with cancer. Ensuring access to innovative anticancer drugs is essential for addressing these disparities and promoting health equity.
2. Economic constraints: Considering the expenditure on cancer care as a high proportion of the overall healthcare budget, developing countries often have limited financial resources and face economic challenges, making it difficult to afford the high costs associated with research, development, and production/import of innovative cancer treatments. Besides, patented medications, which are often prevalent among innovative drugs, might result in high prices, creating financial barriers for both healthcare systems and individual patients.
3. Infrastructure drawbacks: Inadequate healthcare infrastructure in developing countries may impede the effective administration and distribution of novel cancer treatments, affecting the availability of treatment options and overall delivery of care. The absence of specialized healthcare facilities for cancer management is another limitation since access to innovative cancer medications requires access to comprehensive and specialized healthcare facilities.
4. Fragmented healthcare systems: Fragmented healthcare systems may lack the coordination needed to effectively integrate innovative cancer treatments into existing treatment protocols. A holistic approach to healthcare system strengthening is essential for maximizing the impact of these therapies.
5. Regulatory challenges: Resource-limited regions generally face regulatory delays in approving new drugs, impacting the timely availability of innovative anticancer agents. Regulatory processes may lack the required efficiency and capacity to assess and approve the latest treatments.
6. Training gaps: Insufficient education and training of healthcare professionals on administering and monitoring innovative cancer medications can hinder their effective utilization. Training programs must be implemented to ensure the safe and proper use of these therapies.
7. Population-based disparities: Developing countries and regions may have limited participation in clinical trials for innovative cancer treatments, leading to a lack of data on the efficacy and safety of these treatments in diverse populations.
8. Limited research and development (R&D) capacity: Limited research and development capabilities restrict these countries’ ability to significantly contribute to the discovery and production of innovative cancer drugs, creating a dependency on external sources.
9. Public awareness and societal stigmas: Cultural and societal stigmas surrounding cancer can impact early detection, diagnosis, and treatment-seeking behavior. Limited public education about cancer prevention and early detection may contribute to delayed diagnoses and less effective use of available resources, including innovative treatments.
10. Global pharmaceutical market dynamics: The global pharmaceutical market dynamics may prioritize the interests of developed countries, leading to delayed or limited access for developing nations to breakthrough cancer therapies.
11. Ethical considerations: The ethical aspects of conducting clinical trials and introducing new therapies in resource-limited settings require careful attention to ensure patient safety, informed consent, and equitable distribution of benefits.

Cancer knows no borders, and promoting access to novel anticancer therapies contributes to global health security. This process not only facilitates the distribution of anticancer agents, but also establishes a foundation for sustainable healthcare systems in resource-limited countries. Access to innovative medicines improves the patients’ survival and enhances the quality of life for people with cancer due to fewer side effects.

Addressing these obstacles requires a comprehensive and context-specific approach, involving collaborations between governments, international organizations, healthcare providers, and the pharmaceutical industry. Implementing expanded access programs, public–private partnerships, compulsory licensing, patent pooling, and healthcare financing reforms could be considered as some of the initial steps toward these obstacles. Global initiatives should advocate for the availability and access to anticancer agents in developing countries.

By acknowledging and actively working to overcome these challenges, progress can be made in ensuring that patients from developing countries are not deprived of the frontline achievements in the field. Such efforts contribute not only to the well-being of affected individuals but also to the overall advancement of cancer care on a global scale.

## Ethical approval

Not applicable.

## Consent

Not applicable.

## Sources of funding

Not applicable.

## Author contribution

Y.V.-G.: investigation, data curation, and writing – original draft, review and editing; M.-S.H.: conceptualization, data curation, and writing – original draft, review and editing.

## Conflicts of interest disclosure

There are no conflicts of interest.

## Research registration unique identifying number (UIN)

Not applicable.

## Guarantor

Mohammad-Salar Hosseini.

## Data availability statement

No dataset was generated during the study.

## Provenance and peer review

Not commissioned, externally peer-reviewed.
